# Anti-Coagulant and Anti-Thrombotic Properties of Blacklip Abalone (*Haliotis rubra*): In Vitro and Animal Studies

**DOI:** 10.3390/md15080240

**Published:** 2017-08-04

**Authors:** Hafiz Ansar Rasul Suleria, Paul P. Masci, Kong-Nan Zhao, Rama Addepalli, Wei Chen, Simone A. Osborne, Glenda C. Gobe

**Affiliations:** 1 UQ Diamantina Institute, Translational Research Institute, Faculty of Medicine, The University of Queensland, 37 Kent Street Woolloongabba, Brisbane, QLD 4102, Australia; p.masci@uq.edu.au (P.P.M.); k.zhao@uq.edu.au (K.-N.Z.); g.gobe@uq.edu.au (G.C.G.); 2 CSIRO Agriculture and Food, 306 Carmody Road, St Lucia, Brisbane, QLD 4067, Australia; rama.addepalli@csiro.au (R.A.); wei.chen@csiro.au (W.C.); simone.osborne@csiro.au (S.A.O.); 3 Royal Brisbane & Women’s Hospital Campus, University of Queensland Centre for Clinical Research, The University of Queensland, Herston, Brisbane, QLD 4029, Australia

**Keywords:** marine processing waste, blacklip abalone, anti-thrombotic and anti-coagulant activity

## Abstract

Sulphated polysaccharides with anti-thrombotic and anti-coagulant activities have been found in various marine biota. In this study, a previously characterised anti-thrombotic and anti-coagulant extract from blacklip abalone was fractionated by anion exchange chromatography (AEC), pooled (on a sulphated polysaccharide basis) and administered to Wistar rats via oral gavage (N = 8) for assessment as an oral therapeutic. To ensure that the preparation had anti-coagulant activity prior to oral administration, it was assessed in rat blood by thromboelastography (TEG) significantly increasing reaction (R) time (or time until clot formation). Following in vitro confirmation of anti-coagulant activity, 40 mg of the preparation was orally administered to rats with blood samples collected at 2, 4, and 6 h post-gavage. Assessment of all blood samples by TEG showed some prolongation of R time from 355 to 380 s after 4 h. Dosing of the post-gavage blood samples with the abalone preparation to confirm anti-thrombotic activity in vitro revealed residual anti-coagulant activity, further suggesting that oral administration did increase anti-coagulant potential in the collected blood but that bioavailability was low. Assessment of tissues and haematological parameters showed no obvious harmful effects of the abalone preparation in animals. In summary, even though oral administration of fractionated and pooled blacklip abalone extract to rats delayed clotting after 4 h, bioavailability of the preparation appeared to be low and may be more appropriate for intravenous administration as an anti-thrombotic or anti-coagulant therapeutic.

## 1. Introduction

Thrombosis and related disease states are increasing globally and require anti-thrombotic and anti-coagulant therapy. Heparin, one of the most widely available intravenous anti-coagulant therapeutics and the second most abundant, naturally-occurring drug after insulin [1], is a potent anti-coagulant because of its unique binding to anti-thrombin III [2].

Heparin has been the most clinically exploited anti-coagulant for the last 50 years [3]; however, the use of this therapeutic is limited because of its hemorrhagic effect, poor bioavailability, multiple daily dosing, and side effects such as heparin-induced thrombocytopenia (HIT) [4]. Heparin is usually isolated from mammalian sources such as porcine intestinal mucosa and bovine lung, however, isolates from these sources are often comprised of more than one type of glycosaminoglycan that can appear as contaminants with detrimental effects [5]. Also, the disaccharide composition of heparin from different sources displays complexity in sulphation that can result in different coagulation, thrombosis, and bleeding. In a study from Brazil, uncontrolled bleeding occurred when porcine intestinal heparin was replaced by bovine intestinal heparin [6]; even though bovine intestinal heparin displayed half the coagulant activity compared to porcine intestinal heparin, the bleeding side effects were similar. To overcome some of these issues, different low molecular weight heparins, such as Clexane [7], have been developed and modified using an array of techniques including chemical depolymerisation and enzymatic digestion followed by purification targeted to oligosaccharides with high charge densities [4]. 

Alternatives to heparin therapy have been also sought and used to overcome the side effects associated with this therapeutic. The best-known alternative is warfarin; others include lepirudin, argatroban, and bivalirudin [8]. The long half-life and irreversible binding of some of the heparin alternatives, namely lepirudin and argatroban, make the use of these alternatives challenging. Warfarin is a reversible anti-coagulant therapy, but it has similar contraindications for human therapy as heparin. Therefore, alternatives to heparin are still being sought, with a number of new oral low molecular weight anti-coagulants recently approved by the Food and Drug Administration (FDA) including rivaroxaban, dabigatran, apixaban, and fondaparinex (IV; Arixtra). However, these approved formulations all have contraindications that can cause issues in various patients [9].

In the last few decades, there has been a gradual increase in processed marine products with a concomitant increase in waste streams. In 2010, these waste streams were equal to approximately 24 million tonnes. These marine processing streams are also enriched with structurally diverse molecules, especially sulphated polysaccharides that possess a broad panel of bioactivities including anti-coagulant, anti-thrombotic, anti-oxidant and anti-inflammatory activities [10]. Sulphated polysaccharides from marine organisms may provide an alternative source of heparin-like molecules [11]. Marine sulphated polysaccharides may offer some advantages over mammalian heparin, as they show considerably less contamination with viruses and/or prions and have the potential to be useful clinical reagents due to their regular and well-defined structures [12]. An early study in 1977 revealed that there were various glycosaminoglycan (GAG)-like molecules in molluscs [13]. Since then, GAG-like molecules or glycoproteins, have been separated and extracted from molluscs such as pearl oysters and scallops [14]. Abalone is also being investigated as a source of anti-thrombotic and anti-coagulant molecules, as it contains structurally diverse, bioactive components including GAG-like molecules [15]. Recently, several studies have focused on the nutritional and pharmaceutical values of abalone extract. In particular, different types of sulphated polysaccharides obtained from abalone viscera and gonads have shown anti-thrombotic and anti-coagulant activity in vitro [16]. However, in vivo studies are necessary for investigating the suitability of these new molecules as therapeutic candidates. 

To the best of our knowledge, only one study has been published regarding anti-coagulant and anti-thrombotic activity of abalone viscera extract in vivo using rat models [17]. In the proposed research, a preparation from blacklip abalone (*H. rubra*), with confirmed anti-thrombotic and anti-coagulant activity, was provided to Wistar rats by oral gavage. Following oral administration, blood coagulation parameters were assessed to determine if the fractionated abalone extract was bioavailable and whether it could delay clotting time in vivo. 

## 2. Results and Discussion

According to the World Health Organisation (WHO), cardiovascular diseases, including heart disease and stroke related to thrombosis, are the main causes of death globally with predictions that by 2030, almost 3.6 million people will die from these diseases [18]. Heparin has been the most widely used anti-coagulant drug for the last 50 years [19]. Heparin, and other anti-coagulant drugs such as lepirudin and argatroban, have side effects including long half-life, excessive bleeding, and HIT. For these reasons, it is important to look for alternative sources of anti-coagulant agents other than mammalian sources [20].

Marine organisms are increasingly being investigated as sources of bioactive molecules with therapeutic applications as nutraceuticals and pharmaceuticals. In particular, the retrieval and characterisation of these bioactive molecules from marine processing waste contributes valuable information to the vast field of marine natural product discovery [21]. Heparin-like molecules have been identified form various molluscs, and some have less bleeding side effects compared to heparin [22]. Fourteen species from eight families of molluscs contain various sulphated polysaccharides and heparin-like molecules with different disaccharide compositions, molecular weights, and anti-coagulant activities ranging from 5 to 365 IU/mg [23]. The approximate average molecular mass of heparin-like molecules in molluscs is 27,000 Da, and is higher than that of mammalian heparin [24].

In our previous studies, an extract prepared from wild caught blacklip abalone viscera [25,26] using different proteolytic enzymes followed by anion exchange chromatography also showed significant anti-thrombotic and anti-coagulant activity. In vitro prothrombin time (PT), activated partial thromboplastin time (aPTT), and TEG were prolonged by all abalone extracts, with PT increasing in response to anionic fractions. In current research, previously prepared fractions from blacklip abalone extract with confirmed anti-thrombotic and anti-coagulant activity [27] were pooled and provided to Wistar rats by oral gavage.

### 2.1. Freeze Drying of AEC Pool 4 and Anti-Thrombotic Assessment In Vitro

Prior to animal modelling, an aliquot of freeze dried AEC pool 4 was reconstituted and assessed for anti-thrombotic activity in vitro using PT and aPTT assays performed in rat plasma to ensure bioactivity prior to oral administration. As shown in Table 1, freeze drying did not adversely affect bioactivity as evidenced by the significant prolongation of PT and aPTT in rat plasma. In vitro anti-thrombotic activity using PT and aPTT is almost comparable to heparin standard, as we published previously in Suleria et al. [25]. For comparison, the international normalised ratio (INR) was calculated for each PT and aPTT assay using an international sensitivity index (ISI) of 1.2. The INR is used to monitor individuals who are being treated with an anti-coagulant. Overall, both PT and aPTT were significantly prolonged by increasing the sulphated polysaccharide concentrations.

### 2.2. Oral Administration of Fractionated and Pooled Abalone Extract (AEC Pool 4) to Rats and Analysis of Whole Blood

**Figure 1:**
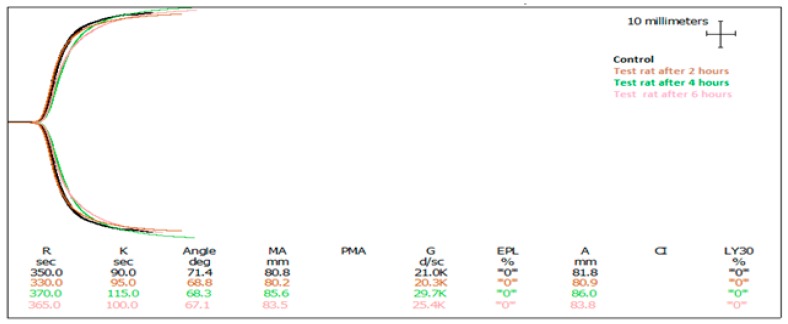
Thromboelastographs of rat blood from control and treatment animals following oral gavage of fractionated and pooled abalone extract (AEC pool 4). The black graph represents the control. The brown, green, and pink graphs show the results of 2, 4, and 6 h post oral delivery, respectively.

Freeze dried AEC pool 4 (40 mg determined on a sulphated polysaccharide basis) in 2.0 mL water was delivered to rats via oral gavage. The dosage was determined by the extrapolation of results from in vitro experiments. In our previous research [26], we concluded that clotting time was delayed by increasing the concentration of sulphated polysaccharides. To determine the oral dose of AEC pool 4, we observed that 25 µg/mL AEC pool 4 (based on sulphated polysaccharide concentration) in blood delayed in vitro clotting by almost two-fold compared to a saline control. Using a predicted bioavailability of 10%, a dose of AEC pool 4 equivalent to 40 mg sulphated polysaccharides was provided to rats with an average body weight of 250 g and a predicted blood volume of 16 mL. Blood samples were taken at one-time point per rat either at 2, 4, or 6 h post-gavage, and subjected to blood hematological, histopathological and TEG. Blood haematology showed no obvious toxicity in control or AEC pool 4 administered groups (Table 2). Tissues including the liver, kidneys, small intestine, and large intestine were also fixed, stained, and examined under a microscope, revealing no obvious changes in morphological characteristics including hydropic change, apoptosis, and necrosis. Subsequently, it appeared that the oral administration of 40 mg AEC pool 4 had no obvious harmful effects on animals in this study. Thromboelastography showed a significant increase in R time in the 4 h post-administration blood samples (Table 3 and Figure 1).

Recently, Li et al. [17] reported that oral gavage of 200–400 mg abalone (*H. discus hannai*) visceral extract/kg rat body weight prolonged aPTT and tail bleeding time (TT) in a dose-dependent manner, but did not increase PT or TT compared to the control group. This study also showed no effect on platelet aggregation or number. Taken together, these results suggest that an increase in aPTT reflects the involvement of the *H. discus hannai* extract in the intrinsic coagulation pathway as opposed to the extrinsic and common pathways that are usually implicated by changes in PT and TT [27,28]. Cui, Wang, and Yuan [14] also reported the beneficial effect of a glycosaminoglycan-like molecule, isolated from the mollusc *Mactra veneriformis*, on deep venous thrombosis in a rat model. In this study, the anti-thrombotic effect was dose-dependent following intravenous administration (0.1, 0.4, and 1.6 mg/kg), resulting in a significant increase in aPTT with no change in PT. Overall, the anti-thrombotic and anti-coagulant effect from *M. veneriformis* was weaker than that of mammalian heparin; however, the bleeding risk was greatly decreased.

### 2.3. In Vitro Anti-Coagulant Assessment of AEC Pool 4 Dosed into Rat Blood

**Figure 2:**
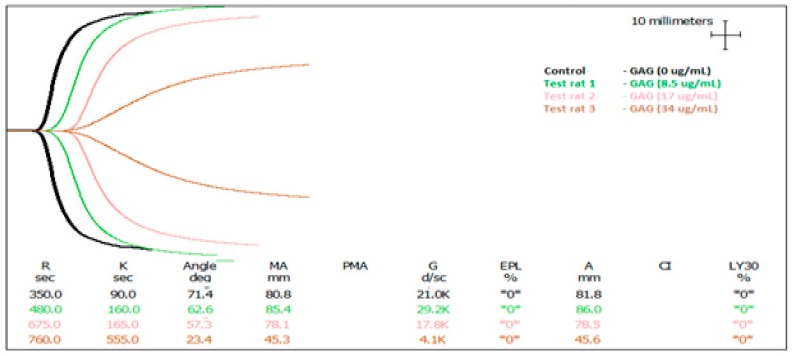
Thromboelastographs of rat blood dosed with fractionated and pooled abalone extract (AEC Pool 4). The black graph represents the control. The green, pink, and brown graphs show blood clotting activity after the addition of 8.5, 17, and 34 µg/mL sulphated abalone polysaccharide, respectively.

Clotting time was only delayed by 25 s in the 4 h post-gavage blood samples. To investigate the lack of anti-coagulant effect achieved following the oral administration of AEC Pool 4, rat blood collected after oral administration was dosed with AEC pool 4 and assessed again by TEG. In Table 4, R time was prolonged significantly by increasing the concentration of AEC pool 4 compared to the control. It is clear from the TEG traces in Figure 2 and the TEG data in Table 4 that AEC pool 4 has anti-coagulant activity in vitro. With respect to the α angle and maximum amplitude (MA) value, AEC pool 4 decreased these parameters significantly as compared to control. Interestingly, the addition of 34 µg/mL AEC pool 4 to blood samples taken from the treatment animals 2, 4, and 6 h post-gavage produced varying R times. Dosing 34 µg/mL AEC pool into blood collected 2 h post-gavage extended R time by 810 s, significantly longer than AEC pool 4 added to blood collected 4 h (720 s, *p* = 0.0151) and 6 h post-gavage (668 s, *p* = 0.002). Clot strength (MA) values and angle followed a similar pattern. This suggests that the oral administration did provide some anti-coagulant and anti-thrombotic molecules that persisted in different levels post-gavage.

The use of marine sulphated polysaccharides as anti-thrombotic drugs appears to be limited to intravenous administration given the high oral dosing required to produce anti-thrombotic and anti-coagulant effects. Some of the new oral anti-thrombotic drugs, such as direct thrombin or factor Xa inhibitors, have overcome this limitation but can still cause bleeding or sometimes unpredictable responses [29]. The oral administration of sulphated polysaccharides is complicated by their size and composition, which makes them resistant to degradation by enzymes produced by vertebrates, or by the bacteria of intestinal flora, reducing intestinal absorption and thus limiting oral administration [30]. An assessment of sulphated polysaccharide concentration in blood following oral administration is needed to determine whether bioavailability is in fact low and whether absorption enhancers such as poly lactic-co-glycolic acid (PLGA) [31], or whether introducing nanoparticle technology [32] or modifications to the sulphated polysaccharides such as depolymerisation and selection of anionic oligosaccharides, may help to overcome the apparent limitations of these molecules as oral therapeutics. 

In finding ways to improve the oral bioavailability of marine sulphated polysaccharides, it must be considered that anti-thrombotic and anti-coagulant activity is not only a consequence of negative charge density, but also varies with the position of sulphated residues and type of constituent sugar (e.g., α-l-Fucp unit or α-l-galactopyranosyl (Galp)) [1]. For this reason, the specific structure of the oligosaccharide fragment of the sulphated polysaccharide that interacts with proteins and other complexes in the blood coagulation system must be characterised. By determining this, efforts can be made to decrease the size of the parent molecule and purify target oligosaccharides in order to produce more bioavailable and active anti-thrombotic and anti-coagulant therapeutics. Special attention must also be paid to the mode of action of these sulphated polysaccharides that will assist in the design of molecules with enhanced bioavailability.

## 3. Material and Methods

### 3.1. Preparation of Fractionated Abalone Extract with Anti-Thrombotic and Anti-Coagulant Activity In Vitro

Australian wild caught blacklip abalone (*H. rubra*) visceral samples were provided by Tasmanian Seafoods, Hobart, Australia. Abalone extract was prepared according to the method described by Suleria et al. [33]. The extract was then fractionated by anion exchange chromatography (AEC), pooled, and de-salted according to the protocol in Suleria et al. [26]. In vitro HCII-mediated thrombin inhibition was measured in all pooled fractions using a kinetic assay as previously described [34], with modifications [35]. In vitro anti-coagulant activity was also confirmed using prothrombin time (PT), activated partial thromboplastic time (aPTT), and thromboelastography (TEG) assays, using previously published methods in Suleria et al. [25]. From all pooled fractions, AEC pool 4 produced the highest anti-coagulant activity as measured by PT, aPTT, and TEG and was selected for in vivo investigations following oral administration.

### 3.2. Freeze Drying of AEC Pool 4

To prepare AEC pool 4 for oral gavage, freeze drying was carried out using a Christ Freeze Dryer EPSILON 2–6D LSCplus (Martin Christ, Osterode am Harz, Germany) by freezing at −50 °C for 4 h prior to drying at −10 °C and 1.03 mbar for 4 h, followed by −5 °C at 1.03 mbar for 12 h, and 0.001 mbar for 6 h.

### 3.3. In Vivo Anti-Coagulant Effect of AEC Pool 4 Following Oral Administration in a Rat Model

Ethics approval was acquired from the University of Queensland Animal Experimentation Ethics Committee (Ethics approval number MED/TRI/519/15/CKDR/MED). Eight Wistar rats (male) were sourced from the Australian Resources Centre (Western Australia), delivered to the Biological Resources Facility of the Translational Research Institute (TRI, Brisbane, Australia), and acclimatised for at least a week prior to experimentation. Rats were housed in the Biological Resource Facility at the Princess Alexandra Hospital, where temperature was maintained at 20 ± 1 °C and humidity kept between 60% and 75% with artificial light for 12 h (7 a.m.–7 p.m.) daily. All animals had unlimited access to food and water. Rats were fully matured with a body weight range of 200–300 g (average 250 g). Oral administration of 40 mg AEC pool 4 (on a sulphated carbohydrate basis as determined by Blyscan Sulfated Glycosaminoglycan Assay (Biocolor Ltd., Carrickfergus, County Antrim, UK)) in 0.2 mL water was performed via oral gavage. The animals were then monitored and one blood sample was taken from each animal at either 2, 4, or 6 h post-gavage (with two animals sampled per time point, *n* = 2). Blood samples were also taken from control animals (*n* = 2) that did not receive any oral extract administrations. 

All animals were anaesthetised with a regimen of 2.3% isofluorane with 2 L per min of oxygen. This regimen was found to provide the deepest anaesthesia over the experimental period. This anaesthetic regimen was developed in past experiments and was found not to interfere with coagulation [36]. After immobilisation, monitoring of anaesthesia levels was carried out through vital signs and paw pressure stress tests. When it was confirmed that adequate anaesthesia was in place, animals were weighed and positioned for surgery for blood collections. The surgical procedure was carried out using aseptic techniques. Blood samples were collected from the posterior vena cava by opening the abdominal cavity of anaesthetized rat and making a V-cut through the skin and abdominal wall 1 cm caudal to the rib cage. For access to the posterior vena cava, normally positioned between the kidneys, intestines were gently shifted to the left and the liver held forward with a gauze pad. An angled 23–25 gauge needle and a 5 mL syringe were used to collect the blood samples. The needle was inserted into the widest portion of the vein and blood was drawn slowly and gently. Three millilitres of blood were taken from each rat at one time point only and collected into 1 mL citrate tubes (3.2% trisodium citrate blood collection tubes, Greiner Bio-One) prior to TEG analysis. 

The animals were then humanely euthanised under anaesthesia. After completion of the experiment, samples of liver, kidney, and small and large intestines were fixed in 4% buffered formalin overnight, then prepared routinely for histology sectioning and staining, for blinded assessment of any pathological changes. Tissue were blocked in paraffin as a tissue array per animal, sectioned at 4 µm, and stained using haematoxylin and eosin. No histological changes were seen with low and high-power microscopy of the sections (data not shown). Blood was either centrifuged to prepare plasma, or was kept as whole blood. Blood coagulation tests were carried out immediately after blood collections.

### 3.4. Histopathological and Hematological Parameters

At the completion of each experiment, samples (*n* = 2) of liver, kidney, and small and large intestines were removed, bisected in an equatorial plane, fixed in buffered formalin at 4 °C and prepared routinely for histopathology. Formalin-fixed tissue was embedded in paraffin using routine methods, and 4 µm sections were cut onto Superfrost Plus histology slides and stained with hematoxylin and eosin (HE; routine histology), as well as periodic acid-Schiff reagent (PAS; identification of glycogen). All HE and PAS-stained tissue sections were viewed using light microscopy and assessed for morphological characteristics, including hydropic change, apoptosis and necrosis, in 10 microscope fields per section at ×200 magnification by the protocol of Wunnapuk et al. [37]. Hematological parameters were measured for each sample (*n* = 1) in the Chemical Pathology Department at the Princess Alexandra Hospital (Brisbane, Australia) by the Jaffe method using a Beckman DxC800 general chemistry analyser (Beckman Coulter, Brea, CA, USA), following the protocol of Korenkova et al. [38].

### 3.5. Statistical Analyses

All statistical analyses were conducted using a one-way ANOVA with Tukey’s multiple and Dunnett’s comparison tests. These calculations were carried out using GraphPad Prism 5 Software for Windows (GraphPad Software, San Diego, CA, USA, www.graphpad.com). Significance was observed at *p* < 0.05.

## 4. Conclusions

In summary, we investigated the anti-coagulant and anti-thrombotic activity of a fractionated and pooled abalone extract following oral administration to rats via gavage. Some anti-coagulant activity was observed 4 h post oral administration. Dosing studies involving post-gavage blood samples revealed residual anti-coagulant activity, confirming the absorption of some abalone molecules following oral administration. Promisingly, there was no evidence of toxicity in blood or tissues following oral administration. These results suggest that abalone extract should be investigated in vivo following intravenous administration. Overall, the present work confirms the in vitro anti-thrombotic and anti-coagulant activity of sulphated polysaccharides from blacklip abalone extract in rat blood, and demonstrates oral bioavailability and anti-coagulant effect in an animal model, albeit low. Taken together, these findings increase the knowledge of potential sources of therapeutic alternatives to heparin. 

## Figures and Tables

**Table 1:** In vitro anti-thrombotic assessment of freeze dried and reconstituted fractionated and pooled abalone extract (AEC pool 4) using prothrombin time (PT) and activated partial thromboplastin time (aPTT) assays performed in rat plasma.

Sample Descriptions	SP (µg/mL)	Time (s)	INR
**PT**			
Rat plasma (Before freeze drying)	0	9.7 ± 0.02	1.0
	50	12.1 ± 0.08 *	1.2
	100	13.3 ± 0.04 *	1.4
	150	15.2 ± 0.01 **	1.6
Rat plasma (After freeze drying)	0	9.1 ± 0.01	1.0
	50	11.2 ± 0.06 *	1.2
	100	12.4 ± 0.08 *	1.4
	150	14.8 ± 0.06 **	1.6
**aPTT**			
Rat plasma (Before freeze drying)	0	31.4 ± 0.3	1.0
	5	39.2 ± 0.7 *	1.2
	10	50.1 ± 0.2 **	1.6
	20	71.5 ± 0.3 ***	2.3
Rat plasma (After freeze drying)	0	30.4 ± 0.01	1.0
	5	38.4 ± 0.2 *	1.3
	10	52.0 ± 0.2 **	1.7
	20	73.8 ± 0.03 ***	2.4

Note: Statistical significance determined using a one-way ANOVA with Dunnett’s Multiple Comparison Test compared to saline control with * *p* < 0.05, ** *p* < 0.01 and *** *p* < 0.001. SP stands for sulphated polysaccharide concentrations and INR stands for international normalised ratio.

**Table 2:** Baseline characteristics of hematological parameters in both control and rats orally administered fractionated and pooled abalone extract (AEC pool 4).

Blood Parameters	Normal Range	Control	Test, 2 h Post-Gavage	Test, 4 h Post-Gavage	Test, 6 h Post-Gavage
White blood cells (10 × 9/L)	5.10–12.16	8.66	11.19	9.2	10.09
Red blood cells (10 × 12/L)	5.79–7.14	6.46	7.54	6.4	7.36
Hemoglobin (g/L)	122–148	135	142	131	139
Hematocrit (Ratio)	0.30–0.50	0.36	0.479	0.37	0.384
Mean corpuscular volume (fL)	53–59	56	63.5	57.8	52.2
Mean corpuscular hemoglobin (pg)	18–22	20	18.8	20.5	18.9
Mean corpuscular hemoglobin concentration (g/L)	330–410	370	296	354	362
Platelet count (10 × 9/L)	600–700	700	603	641	680
Platelet large cell ratio (%)	3.8–4.5	4.1	4.6	3.6	4.4
Platelet count (%)	0.4–0.6	0.47	0.52	0.42	0.45

Note: ranges obtained from Wistar Rat—Charles River Laboratories: http://www.criver.com/files/pdfs/rms/wistar-rats/rm_rm_r_hematology_crl_wi_br_sex_age.aspx, http://www.doiserbia.nb.rs/Article.aspx?id=0354-46640903353B#.V4IL0fl96M8.

**Table 3:** Assessment of anti-coagulant activity by TEG in rat blood samples following oral administration of fractionated and pooled abalone extract (AEC pool 4).

Sample Description	SP (mg)	R (Sec)	Angle (α)	MA (mm)
Control	0	355 ± 5	66.1 ± 2.2	71.9 ± 13.7
Test 1—2 h post gavage	40	335 ± 7.5	67.1 ± 1.2	83.5 ± 14.7
Test 2—4 h post gavage	40	380 ± 10 *	71.1 ± 3 *	60.1 ± 2.4
Rat 3—6 h post gavage	40	360 ± 15	80.2 ± 0.2 **	75.1 ± 14.5

Note: Results are expressed as the mean of two animals (*n* = 2) ± standard deviation. Statistical significance determined between control and test animals using unpaired *t*-tests with * *p* < 0.05 and ** *p* < 0.01. (SP stands for Sulphated polysaccharides concentration and MA stands for maximum amplitude).

**Table 4:** In vitro anti-coagulant assessment of fractionated and pooled abalone extract (AEC Pool 4) dosed into rat blood.

Sample Description	SP (µg/mL)	R (Sec)	Angle (α)	MA (mm)
Control	0	375 ± 5	66.1 ± 2.2	71.9 ± 13.7
Test 1—2 h post-gavage	8.5	427 ± 2.2 **	64.2 ± 4.1	69.8 ± 7.5
	17	552.5 ± 3.5 ***	62.25 ± 6.6	57.1 ± 10.1
	34	810 ± 5 ***	29 ± 1 **	37.75 ± 3.4 **
Test 2—4 h post-gavage	17	357.5 ± 7.5	70.65 ± 0.7	67.35 ± 13.4
	34	720 ± 15 **	28 ± 4.6 **	52.4 ± 7.1 *
Test 3—6 h post-gavage	17	467.5 ± 12.5 *	64.85 ± 2.2	73.65 ± 10.8
	34	667.5 ± 7.5 ***	50.4 ± 6.9	72.85 ± 5.2
	50	No blood clot		

Note: Results are expressed as the mean of two animals (*n* = 2) ± standard deviation. Statistical significance determined between control and test groups using unpaired *t*-tests with * *p* < 0.05, ** *p* < 0.01, and *** *p* < 0.001. No blood clot indicated that no clot formation was observed at 50 µg/mL sulphated polysaccharide.
